# Loss of Livelihood, Wages, and Employment During the COVID-19 Pandemic in Selected Districts of Chhattisgarh in India, and Its Impact on Food Insecurity and Hunger

**DOI:** 10.3389/fpubh.2022.810772

**Published:** 2022-05-06

**Authors:** Angeline Jeyakumar, Devishree Dunna, Mitravinda Aneesh

**Affiliations:** ^1^School of Health Sciences, Savitribai Phule Pune University, Pune, India; ^2^School of Hospitality and Tourism, University of Johannesburg, Johannesburg, South Africa; ^3^Department of Nutrition and Dietetics, Mount Carmel College, Bengaluru, India

**Keywords:** food security, migration, tribal, non-tribal settings, loss of livelihood

## Abstract

The COVID-19 pandemic has exacerbated the existing food insecurity in developing nations. The cumulative effect of restricted mobility to curtail the spread of the infection, loss of livelihood and income, worst affected the economically weaker sections. Our work examined the availability, accessibility, and affordability of food during the first wave of the pandemic using the FAO, HFIAS questionnaire, in a random sample (*N* = 401) from Kanker and Narayanpur districts in Chattisgarh, an Empowered Action Group state, in India. Total food security scores were derived by summing the individual scores. Percentages above and below the median scores were used to assess food insecurity. Proportion Z test was used to compare settings and a generalized linear model was used to determine the association between dependent and independent variables. Of the 63% non-tribal population, a greater percent experienced income loss (13.4%) and worried about not having sufficient food (40%). A significantly higher proportion from the non-tribal regions reported food scarcity in the household (34%) and experienced hunger (15%). Non-tribal participants (77%) scored ≤ median (score 8) demonstrating high food insecurity. The odds of poor food access increased in the non-tribal settings (B: 0.024, 95% CI: 0.011–0.051, *P* < 0.001), income between Rs. 10,000–29,000/- per month (B: 0.385, 95% CI: 0.146–1.014, *P* < 0.05) and among those experiencing total or partial income loss (B: 0.505, 95% CI: 0.252–1.011, *P* < 0.05). Urban residence increased the odds of poor food availability (B: 15.933, 95% CI: 3.473–73.096, *P* < 0.001). Being male (B: 0.450, 95% CI: 0.208–0.972, *P* < 0.05), and not experiencing income loss (B: 0.367, 95% CI: 0.139–0.969, *P* < 0.05) decreased the odds of poor availability and affordability (B: 0.153, 95% CI: 0.067–0.349, *P* < 0.001). Non-tribal setting increased the odds of poor affordability (B: 11.512, 95% CI: 5.577–23.765, *P* < 0.001) and hunger (B: 19.532, 95% CI: 7.705–49.515, *P* < 0.001). Being male (B: 0.445, 95% CI: 0.277–0.715, *P* < 0.05) and higher age (B: 0.936, 95% CI: 0.936–0.906, *P* < 0.001) decreased the odds of food insecurity as per the total food security score. While India is likely to experience multiple waves, actions urgent and targeted toward the needs of the vulnerable sections be prioritized to endure and overcome the impact of the pandemic.

## Introduction

The COVID-19 pandemic, one of the greatest crises of the last decade aggravated the existing food insecurity predicaments globally. The United Nations has predicted that an additional 130 million would suffer acute food insecurity, more concentrated in the developing nations (1). In India, the lockdown imposed by the government, to control the pandemic during the first wave led to the down sliding of the Indian economy with seemingly lasting effects on the prevailing nutritional situation (2). Before India recovered from the first wave, the second wave hit with greater intensity. The impact of this multi-fold effect of pandemic leaves India with incalculable consequences, further impeding the achievement of the development goals. As the country experiences an economic slowdown, employment and income losses have driven populations to the brink of poverty (3, 4).

Poverty combined with lockdown extended over long periods worsened food insecurity. Transport restrictions, disruption in the supply chain, and shortage of manpower hamper the production, storage, and distribution of food (5). Consequently, food shortage, escalation of food prices, alongside the loss of livelihood, wages, and employment were experienced across the income groups (6). The dire consequence of these events, escalated the problem of hunger in India, its impact much experienced by the vulnerable in the population; the poor, daily wage laborers, and those employed in the unorganized sectors (7). The first wave witnessed the discontinuation of the supplementary feeding program and school mid-day meal program that contribute to food and nutrition security among the lower-income groups' (6). Pre-COVID statistics of hunger and hidden hunger reflect in child undernutrition (stunting 35%, underweight 32%) and anemia among children and pregnant women [67 and 52% respectively (8)] which is likely to worsen during the pandemic.

In a vast nation with varying degrees of economic uncertainty and food insecurity in different states, the food supply, and value chain vary in different regions and the impact of this pandemic would conceivably be varied in urban, peri-urban, and rural settings (9). It is, therefore, worth exploring the differences in impact, as the lockdown too was implemented with varied stringency in different settings.

Chhattisgarh being an Empowered Action Group (EAG) state is slow in the economic and demographic transition. The geography and demography of Chhattisgarh account for its limited progress that reflects in its 14th position out of 17 Indian states as per the hunger index (10). Almost 77% of the total Chhattisgarh population lives in rural areas and 10% of the total Indian tribal population resides in Chhattisgarh (11). With the already prevailing food-insecure situation, Chhattisgarh was the first state in India to introduce the food security act in December 2012 (12).

In Chhattisgarh, agriculture and engagement in daily labor are the chief sources of income (13, 14). They mostly depend on the public distribution system and mid-day meals, and the Anganwadi (Government preschool centers) plays an important role in maintaining the nutritional requirements of pregnant women and children. Despite the food security policy and programs in place, the maternal and child health indicators are fairly poor, especially in rural and tribal regions. Undernutrition among children below 5 years is higher in rural (39.6%) than in urban regions (30.2%) [NFHS 4, (15)]. In the absence of National prevalence of undernutrition in the tribal regions, regional studies reflect a high prevalence that ranges from 54.7 to 82% (16, 17).

Lessons from the HIV pandemic predict a post-pandemic upsurge in undernutrition and child mortality as a consequence of hunger (18). The impact of this pandemic on food security in Chhattisgarh is worth studying, as the indirect effects of the pandemic perhaps will worsen its maternal and child health indicators. There is little evidence about the prevailing condition of food insecurity during this crisis in EAG states. Studying hunger at the backdrop of income or livelihood loss during a pandemic is vital to plan appropriate interventions and rethink public health policies for emergency preparedness specifically in these regions. The present study aims to assess food accessibility, affordability, and availability in different settings of Chhattisgarh and determine the factors of food insecurity during the COVID-19 pandemic.

This work was motivated by the global effort to study food access and security during the COVID-19 crisis with the international task force (19).

## Methods

### Study Design and Setting

A cross-sectional survey was conducted between November 2020 and January 2021 from urban, rural, and tribal regions of Chhattisgarh. Of the 28 states and 7 union territories in India, 8 states are referred to as EAG states. EAG states experience slow socioeconomic and demographic transition and also fare poorly in health indicators. Chhattisgarh is among one of the EAG states located in East-Central India. The greater percent of the Chhattisgarh population reside in rural and tribal settings, about one-third of the population is tribal and 80% of the population resides in the rural regions and fare poorly in health indicators. For the present study, the rural and tribal data were collected from two villages of Kanker and Narayanpur districts, respectively, situated in the south of Chhattisgarh. From these districts f, two villages, Selegaon and Gudadi from Kanker and Narayanpur districts were selected for convenience and ease of access during the pandemic.

### Sample

Considering a prevalence of 21% of diet diversity among children under-five as a proxy indicator of food insecurity, from the Comprehensive Nutrition Survey (2018–19) (20), at 95% CI, 5% precision, 1.5 design effect, and 10% non-response, the estimated sample size was *N* = 420. Thus a random sample of 420 respondents was enrolled in the study. Respondents who were above 18 years of age and who consented to participate in the study were recruited. Of the 420 samples, a usable 401 questionnaires that had complete data were considered for the study.

### Data Collection

Data was collected by researchers trained in public health nutrition research techniques. They were aware of the objectives and ethical procedures to adhere to this study. The study is part of a global food access survey that employed online data eliciting procedures (19). However, to study food insecurity in Chhattisgarh, which involved data collection in rural and tribal regions we conducted this study through face-to-face interviews. The list of households covered by the Anganwadi centers was obtained from the Anganwadi workers and the data was collected by household visits.

### Tools and Techniques

A modified version of the Household Food Insecurity Access Scale (HFIAS) developed by the Food and Agricultural Organization (21) was used to elicit information about the availability, accessibility, and affordability of food during the pandemic. The questionnaire was translated to the Hindi language. It was pretested to check the flow of questions and usage of relevant terminologies. Each interview lasted for 20 and 45 min.

### Variables

The HFIAS questionnaire elicited information on the dependent variables that included availability, accessibility, affordability of foods, and experience of hunger. Independent variables included socio-demographic characteristics such as age, gender, education, loss of employment, or livelihood. The respondents answered the questions for the household.

### Ethical Consideration

The study was approved by the Institutional Ethics Committee (Ref: SPPU/IEC/2020/83). Participants were briefed about the study and written consent was obtained before the interview and confidentiality of data was ensured. The respondents were free to withdraw from participating in the survey at any point during the interview.

### Data Analysis

Data were entered cleaned and coded in excel and then imported to Statistical Package for the Social Sciences (SPSS, NY: IBM Corp version 20) for analysis. Descriptive statistics were used to describe the study population. Food security was evaluated by deriving food security scores from the variables selected from HFIAS (21). Food access [2 questions, MPS = 10] was scored using the Likert scale where the responses were scored from one to five, the highest score indicated poor access to food. Food availability [maximum possible score (MPS) = 6], affordability [MPS = 4], and hunger [MPS = 3] scores were derived from dichotomous responses where a positive response of food insecurity experience scored one and a negative response scored zero. Proportion *Z*-test was used to test for differences in proportions between non-tribal and tribal settings. A generalized linear model was used to determine the association between the variables.

## Results

### Sociodemographic Characteristics

Table 1 shows the distribution of socio-demographic characteristics. Among the total respondents, over 60% represented the non-tribal settings. Almost 60% of the respondents were females and the mean age of the respondents was 30.11 years (SD ± 9.77). Almost three fourth (74.8%) of participants received secondary education and over 60% [247 (61.6%)] were married. Nearly 90%, [355 (88.5%)] participants reported having less than two children. Almost 70% [283 (70.6%)] reported a family income of less than Rs. 10,000 per month. And <20% each reported a family income category between Rs. 10,000, 29,000, and >30,000, respectively.

**Table 1:** Distribution of socio-demographic characteristics.

**Variables**	**Frequency (*n =* 401)**	**Percentage (%)**
**Setting**
Non-tribal	253	63.1
Tribal	148	36.9
**Gender**
Male	172	42.9
Female	229	57.1
**Mean age (years)**	30.11 ± 9.771	
**Education**
Primary	101	25.2
secondary	300	74.8
**Marital status**
Married/ Co-habiting	247	61.6
Single/ Divorced	154	38.4
**Number of children**
<2	355	88.5
>2	46	11.5
**Income**
<10,000	283	70.6
10,000–29,000	72	18.0
>30,000	46	11.5

### Comparison of Food Security in Different Settings

Table 2 shows the differences in food security indicators between tribal and non-tribal settings during the pandemic. We used income loss or income uncertainty as proxy indicators to study *affordability*. A greater percent of non-tribal respondents reported having experienced income loss (non-tribal 13.4% vs. tribal 3.4%) and experienced fear of income loss (Non-tribal 27.7% vs. Tribal 4.7%) during the pandemic. With regards to *access to food*, a significantly greater percentage of the respondents from non-tribal (56.1%) regions reported having visited local markets more than three times in a week than in the tribal regions (4.1%). A significantly higher proportion [>60%] of tribal respondents reported to have visited the local market once and another 29.7% never visited the market. About 90–97% of respondents from both settings reported having never consumed food from outside services. The results need to be carefully interpreted as markets in tribal settings often operate weekly and therefore cannot be interpreted as having poor or less access to food. Concerning household *food availability*, a significantly higher percentage of urban and rural respondents (41.9%) were worried about not having enough food to eat than those from tribal settings (6.8%). A significantly higher proportion of urban and rural respondents experienced the inability to eat the preferred food (40%), had access to a limited variety of foods (Non-tribal 39.5 vs. Tribal 5.4%), and ate smaller meals than the tribal respondents (Non-tribal 22.1 vs. Tribal 3.4%), while the tribal respondents had significantly more access to free food (41.9 vs. 12.3%). Similar was the reported experience of hunger, where close to 15% of the non-tribal regions remained hungry during the day and or night and 34% did not have food in the household which was higher than the tribal households and these differences were significant (*p* = 0.05).

**Table 2:** Distribution of reported experiences of food insecurity during the pandemic.

**Variables for food security**	**Non-tribal (%)**	**Tribal (%)**
**1. Affordability: Income loss or insecurity as proxy indicators**		
**for affordability**		
**Loss of income**		
Yes	19	3.4
No	81	96.6
**Worried about losing income**		
Yes	27.7	4.7
No	72.3	95.3
**2. Accessibility**		
**Visited local weekly markets** ^*^		
More than three times	56.1	4.1
Three times	7.5	1.4
Twice	12.3	4.1
Once	16.2	60.8
Never	7.9	29.7
**Consumed food from outside**		
More than three times	1.6	1.4
Three times	2.4	0.0
Twice	3.6	0.0
Once	3.6	1.4
Never	88.9	97.3
**3. Availability**		
**Worried about not having enough food**		
Yes	41.9	6.8
No	58.1	93.2
**Not able to eat kind of food preferred**		
Yes	43.1	8.1
No	56.9	91.9
**Had to eat a limited variety of food**		
Yes	39.5	5.4
No	60.5	94.6
**Had to eat some food that you did not want to eat**		
Yes	34.8	6.8
No	65.2	93.2
**Had to eat a smaller meal than you felt you needed**		
Yes	22.1	3.4
No	77.9	96.6
**Got free donated food**		
Yes	12.3	41.9
No	87.7	58.1
**4. Reported experience of hunger**		
**No food to eat of any kind** ^*^		
Yes	34.8	4.1
No	65.2	95.9
**Went to bed hungry** ^*^		
Yes	14.2	2.0
No	85.8	98.0
**Remained hungry both during day and night** ^*^		
Yes	16.6	0.7
No	83.4	99.3

* *P ≤ 0.05*.

Table 3 shows the comparative scores of food security indicators between settings. A greater percentage of non-tribal participants (77%) scored ≤ median (8) representing high food insecurity and 88.5% of tribal respondents scored above the median (9) indicating better food security.

**Table 3:** Median scores of different components of food insecurity.

**Scores of food security domains**	**Non-tribal (%)**	**Tribal (%)**
Accessibility^*^(MPS 10)
< =median (8)	77.1	11.5
>=9	22.9	88.5
Availability^*^(MPS 6)
< =median (1)	70.5	98.5
2	29.5	1.5
Affordability^*^(MPS 4)
< =median (0)	41.9	91.9
>median (1)	58.1	8.1
**Hunger** ^*^(MPS 3)
< =median (0)	55.7	95.9
>median (1)	44.3	4.1
Total food insecurity score^*^(MPS 22)
< =median (10)	50.6	73.6
>median (11)	49.4	26.4

* *MPS, Maximum Possible Score*.

### Determinants of Food Insecurity During the Pandemic

Table 4 shows a generalized linear model that was used to examine the association of background characteristics with the food insecurity scores. Socio-demographic characteristics were tested with food accessibility scores in model 1, availability score in model 2, affordability in model 3, hunger in model 4, and model 5 with total food security score.

**Table 4:** A generalized linear model of background characteristics and score of food insecurity.

**Factors**	**Model 1**				**Model 2**				**Model 3**				**Model 4**				**Model 5**			
	**Food accessibility score**				**Food availability score**				**Food affordability score**				**Hunger score**				**Total food insecurity score**			
	**B**	**Exp. B**	**95% CI**		**B**	**Exp. B**	**95% CI**		**B**	**Exp. B**	**95% CI**		**B**	**Exp. B**	**95% CI**		**B**	**Exp. B**	**95% CI**	
**Settings**																				
Non-tribal	−3.735	0.024^**^	0.011	0.051	2.768	15.933^**^	3.473	73.096	2.443	11.512^**^	5.577	23.765	2.972	19.532^**^	7.705	49.515	0.603	1.28^**^	1.02	3.251
Tribal																				
**Age**																	−0.067^**^	0.936	0.93	0.90
**Gender**																				
Male					−0.799	0.450^*^	0.208	0.972									−0.809	0.445^*^	0.27	0.71
Female																				
**Income (INR)**																				
<10,000									0.871	2.390^*^	1.106	5.162								
10,000–29,000	−0.955	0.385^*^	0.146	1.014					1.039	2.825^*^	1.179	6.771								
>30,000																				
**Income lost**																				
Yes																				
No	−0.684	0.505^*^	0.252	1.011	−1.003	0.367^*^	0.139	0.969	−1.880	0.153^**^	0.067	0.349	−0.740	0.477^*^	0.251	0.908	−1.357	0.257^*^	0.12	0.52

(* *P < 0.05,*

** *P < 0.001); Reference is < = Median*.

In model 1, setting, income, and income losses were associated with food accessibility scores. The respondents from non-tribal settings had 0.024 times less access to food (B: 0.024, 95% CI: 0.011–0.051, *P* < 0.001). The respondents with income between Rs. 10,000 and 29,000/month had 0.38 times more access to food (B: 0.385, 95% CI: 0.146–1.014, *P* < 0.05). The participants who did not lose either part or full source of income had 0.50 times more access to food during a crisis (B: 0.505, 95% CI: 0.252–1.011, *P* < 0.05).

In model 2, setting, gender, and income lost showed a significant association with food availability scores. The residents of the urban settings in Chhattisgarh showed 15.93 times higher odds of poor food availability as compared to their rural counterparts (B: 15.933, 95% CI: 3.473–73.096, *P* < 0.001). Between gender, males experienced 0.45 times fewer concerns related to food availability compared to women (B: 0.450, 95% CI: 0.208–0.972, *P* < 0.05). The respondents who did not lose their income were 0.367 times less likely to face issues related to the non-availability of food (B: 0.367, 95% CI: 0.139–0.969, *P* < 0.05).

In model 3, settings, family income and income lost showed significant association with affordability score. The non-tribal residents showed an 11.51 higher odds of poor affordability score (B: 11.512, 95% CI: 5.577–23.765, *P* < 0.001). Respondents with family income < INR. 10,000/- showed 2.39 times higher odds of poor affordability (B: 2.390, 95% CI: 1.106–5.162, *P* < 0.05), whereas income between INR10,000 and 29,000 faced 2.82 times lesser odds of poor affordability (B: 2.825, 95% CI: 1.179–6.771, *P* < 0.05). The respondents who never lost their income during the COVID-19 crisis showed 0.153 times lesser odds of poor affordability (B: 0.153, 95% CI: 0.067–0.349, *P* < 0.001).

In model 4, settings and income losses were significantly associated with hunger scores. The non-tribal respondents faced 19.53 times more hunger than those in tribal regions (B: 19.532, 95% CI: 7.705–49.515, *P* < 0.001), and the population who never lost their income have 0.477 times experienced less hunger than those who have lost their income (B: 0.477, 95% CI: 0.251–0.98, *P* < 0.05).

In model 5, settings, age, gender and income lost showed significant association with total food insecurity score. The population residing in the non-tribal area was 1.28 times more food insecure during the pandemic than those in the tribal regions (B: 1.28, 95% CI: 1.028–3.251, *P* < 0.001). It was found that as age increased there are 0.936 times lesser odds of food insecurity (B: 0.936, 95% CI: 0.936–0.906, *P* < 0.001). Men were 0.445 times less food insecure than women (B: 0.445, 95% CI: 0.277–0.715, *P* < 0.05) and those who never lost a part or full source of income during the crisis were 0.477 times less food insecure than those who lost their income (B: 0.477, 95% CI: 0.251–0.908, *P* < 0.05).

## Discussion

This work was an attempt to study food insecurity in Kanker and Narayanpur districts of Chhattisgarh during the lockdown period. A state categorized as EAG is likely to have experienced varying levels of food insecurity and its consequences during the extended lockdown. In India, with the rising number of infections, the fear of another lockdown is experienced by the population. This work is therefore important as it highlights the prevailing conditions of income loss and the consequent hunger experienced which is likely to worsen in an EAG state.

### Loss of Livelihood, Migration, and Food Insecurity

During the pandemic, loss of employment, daily wages, or income in any form was experienced across settings. This included the urban poor who are often migrants from rural or tribal settings who are daily wage laborers, and those who represent the middle and upper-income groups. With income loss or financial insecurity as the context during the pandemic, we studied the four domains of food insecurity viz. accessibility, availability, affordability, and hunger during the pandemic. India, state-wise data on food security in the pre-COVID era are unavailable as per the core indicators (Accessibility, Availability, and Affordability). However, comprehensive data on direct and proxy indicators are available from the Food and Nutrition Security Analysis (FNSA), India (22), and the NFHS 4 (2015-16) (15). The per-capita expenditure on food between 2011 and 12 in the rural and urban Chhattisgarh was 45.1 and 78.8% respectively (23). Chhattisgarh was among the four states that showed a decline in protein intake with a per-capita per-day intake lower than the RDA of 48gm. It is the only state where the protein intake was less, both in the 2004–5 and 2011–12 statistics. Concerning energy, between 2004–5 and 2011–12, per-capita per-day intake increased in most states of India. On the contrary, 11 states including Chhattisgarh showed declining trends during this period. Fat intake too was lower than RDA andower intake was significant among SC and ST (24).

Loss of income and fear of income loss together reported by nearly 50% points to the gravity of economic insecurity experienced. A higher percent from the non-tribal regions reported economic loss and instability. In the absence of core indicators, indirect indicators from the NFHS 4, indicate an infant mortality rate (IMR) of 54%, and an under-five mortality rate (U5MR) of 64%. Prevalence of stunting was 31.6 and 39.2, wasting was 20.6 and 23.7 and under-weight was 30.2 and 39.6 percent in the rural and urban regions respectively (24). These figures indicate high prevalence in urban settings. Also, our findings reveal that the population from non-tribal settings in Chhattisgarh faced more food insecurity than the tribal regions, similar to the pre-COVID literature, which reported that food insecurity is higher in urban and rural settings than in isolated settings of India (25–27). Due to migration from rural and tribal settings to urban regions for better livelihoods, they face serious challenges to meet the basic requirements (28, 29) in addition to the loss of livelihood during the pandemic. While the pandemic has worsened the situation, these settlements have been projected to increase by 2050 as food insecurity and poverty are already prevailing in the isolated regions (30). It is well known that food security prevails in the tribal regions, but the urban poor has become the “new hungry” due to the pandemic. This could contribute to a net increase in the proportion of the population who are hungry deviating further from the sustainable development goals. It is thus clear that poverty increases the risk of hunger irrespective of setting.

### Access to Food in Different Settings During the Imposed Lockdown

We explored the accessibility to food by studying the access to local markets and the frequency of consuming food from outside sources, we observed that the tribal population visited local markets and consumed foods prepared outside than homes less frequently. However, these should be interpreted with caution as we cannot conclude that they have restricted food access during the pandemic as the chief occupation of tribal people is agriculture which is a product yielding activity that results in agricultural produce (31) and their dependence on markets and shops for their livelihood is minimal. Markets are often weekly and therefore weekly access as an indicator of food access may not be the right indicator for tribal settings.

Better food access need not necessarily indicate food security. The stringent 21 days lockdown which further extended to 60 days affected the availability of the food in non-tribal settings. The disruption in the supply chain perhapsbe the led to the unavailability of food in urban settings. This has been documented in other studies where the lockdown disrupted transportation and supply networks, induced labor shortage, fuelling a panic situation that brought about the hoarding of food items which further increased the burden on the demand side (32, 33).

Although the majority of the study participants did not require food assistance, almost 42% from the tribal setting have reported having received help from family and friends which portrays the sharing culture of the tribal population that could have contributed to better food security whereas the non-tribal population majorly depend on the public distribution system (PDS), which suffers from disrupted supply chain that prevents optimal functioning during a pandemic. Although our study identified non-tribal residents consumed fewer or skipped meals due to lack of money, other studies reported similar situations in the tribal region (34). We found. The difference in observations in the tribal and non-tribal settings could have been the dependency on farming, fisheries, and hunting in the tribal regions. Also, a majority of our study population reported a low-income level per month, which likely is to have contributed to this observation. Various mathematical models have projected public health strategies such as masks, social distancing, and media for behavior change (35). Identification of strategies to improve income security or prevent financial setbacks is a critical need to address as it is projected that the virus will become endemic and seasonal (36, 37). Such models are therefore essential to project food and income security situations.

The study identified the factors contributing to food insecurity during the pandemic. Our analysis suggested that residents of the non-tribal areas, who lost their income during the COVID-19 crisis, women and young people who represent the production section of the population were those affected with high food insecurity scores in Chhattisgarh. Evidence of vulnerability of women to poverty and high propensity of migration among the young in Chhattisgarh exist (38). Similar experiences of food insecurity leading to hunger due to the restrictions imposed, in urban regions have been reported by other studies in India and its neighboring countries (33, 39). The results were consistent that in the pre-COVID times where food insecurity was more prevalent among households with lower monthly income especially among women and children (34, 40).

### Limitations

Despite capturing the seriousness of food insecurity our work had several limitations. Due to restrictions, we studied selected areas which limited the generalization of our findings. Vulnerable populations such as pregnant women and households with children could have faced varying levels of food security and our sample and analysis did not consider these specific population groups. Data on food groups that were not elicited in our work limited assessment of diversity and pattern of foods consumed in different settings of Chhattisgarh. The results of our study from tribal settings need to be carefully interpreted as availability of food may already be a concern and implementation of lockdown would not have been stringent in these settings. Further, the loss of income in the urban and rural settings was much more in our study as compared to tribal regions. It is also likely that income loss in tribal regions would have yielded limited responses as income in tribal regions need not always be in the form of cash. Gainful activities leading to gaining agricultural or farm or forest produce are also considered as income (41) and this was not elicited in this study. Further, we have not considered the exposure to the virus in these settings that would have added to the multiple burdens. The second wave affected the rural and tribal regions severely more than the first. Therefore, the findings are limited to the experiences during the first wave.

## Conclusion

The unprecedented crisis of COVID-19 has worsened the existing problem of food insecurity, especially in urban Chhattisgarh. To address this situation nutritional programs must run uninterruptedly in previously vulnerable territories. Emergency feeding programs extended to all age groups would be an immediate response and financial support to the vulnerable population can increase the affordability of food to reduce hunger and prevent undernutrition. Long-term strategies should be planned based on lessons learned from this pandemic, this would be the first step for preparedness for future disasters.

## Data Availability Statement

The raw data supporting the conclusions will be made available by the authors on request.

## Ethics Statement

The study was approved by the Institutional Ethics Committee (Ref: SPPU/IEC/2020/83). Participants were briefed about the study and written consent was obtained before the interview and confidentiality of data was ensured. The respondents were free to withdraw from participating in the survey at any point during the interview.

## Author Contributions

AJ and MA conceptualized the study. DD collected data and performed the analysis under the guidance of AJ and MA. The first manuscript was written by AJ and edited by all authors.

## Conflict of Interest

The authors declare that the research was conducted in the absence of any commercial or financial relationships that could be construed as a potential conflict of interest.

## Publisher's Note

All claims expressed in this article are solely those of the authors and do not necessarily represent those of their affiliated organizations, or those of the publisher, the editors and the reviewers. Any product that may be evaluated in this article, or claim that may be made by its manufacturer, is not guaranteed or endorsed by the publisher.

## Abbreviations

EAG: Empowered Action Group
HFIAS: Household Food Insecurity Access Scale.
